# Updated clinical management guidance during the COVID-19 pandemic: thyroid nodules and cancer

**DOI:** 10.1530/EJE-21-0716

**Published:** 2022-01-24

**Authors:** Evanthia Giannoula, Ioannis Iakovou, Luca Giovanella, Alexis Vrachimis

**Affiliations:** 1 Academic Department of Nuclear Medicine, University Hospital AHEPA, School of Medicine, Aristotle University of Thessaloniki, Thessaloniki, Greece; 2 Academic Department of Nuclear Medicine, General Hospital Papageorgiou, Aristotle University of Thessaloniki, Thessaloniki, Greece; 3 Clinic for Nuclear Medicine and Competence Centre for Thyroid Diseases, Imaging Institute of Southern Switzerland, Bellinzona, Switzerland; 4 Clinic for Nuclear Medicine, Zurich University Hospital, Zurich, Switzerland; 5 Department of Nuclear Medicine, German Oncology Center, University Hospital of the European University, Limassol, Cyprus; 6 C.A.R.I.C. Cancer Research & Innovation Center, Limassol, Cyprus

## Abstract

Healthcare settings, including nuclear medicine (NM) departments, promptly adjusted their standard operating procedures to cope with the unprecedented crisis caused by coronavirus disease 19 (COVID-19) pandemic. Nuclear thyroidology has adopted changes and predicated on a careful risk–benefit analysis, in order to prevent a potential spread of the virus while being at the same time effective, safe and preserving their quality of essential services. Since most thyroid nodules (TNs) are benign, and malignant neoplasms are characterized by an indolent natural history, it is generally safe to delay diagnostic and therapeutic procedures. In this respect, the main adjustments that nuclear thyroidology has adopted are summarized into the following: general workplace adjustments including remote work for NM staff; postponing appointments for consultation, diagnostic and therapeutic purposes and rescheduling based on individualized risk stratification; telemedicine; preparation for possible issues on radiopharmaceuticals synthesis and delivery; preventing measures and protocols to minimize or avoid potential COVID-19 infection of patients and medical staff. This document should be considered as updated guidance on how clinical management of TNs and thyroid cancer has been altered, remodeled and adapted to the new circumstances in the COVID-19 era, based on the rapidly growing volume of scientific information regarding the new coronavirus.

The current paper is an update of the Clinical Management Guidance during the COVID-19 Pandemic: Thyroid nodules and cancer (1). Since the World Health Organization declared the novel coronavirus disease 19 (COVID-19) a global pandemic in March 2020, the global health crisis has changed nearly everything, affecting both economies and societies globally and causing unprecedented pressure on healthcare systems worldwide. The major change that has taken place, compared with the previous iteration of the guidance, is the development of COVID-19 vaccines (https://www.scmp.com/news/china/society/article/3103121/coronavirus-who-backed-chinas-emergency-use-experimental, Accessed: June 16, 2021), as well as the release of numerous treatment Guidelines (https://www.ncbi.nlm.nih.gov/sars-cov-2/. Accessed: June 13, 2021, https://www.covid19treatmentguidelines.nih.gov/about-the-guidelines/guidelines-archive/. Accessed: June 20, 2021).

At the same time, healthcare settings, including nuclear medicine (NM) departments, promptly adjusted their standard operating procedures to cope with the pandemic cases and deliver their services. Several reports have been issued to serve as guidance on all aspects of NM practice, including nuclear thyroidology (1, 2), with changes being predicated on a careful risk–benefit analysis, in order to prevent a potential spread of the virus while being at the same time effective, safe and preserving their quality of essential services. Elective treatments, screening and diagnostic procedures are recommended to be postponed and rescheduled, canceled or replaced with alternative procedures, to avoid exposing patients and medical staff to unnecessary risks (3, 4, 5).

The risk for serious disease and death in COVID-19 cases increases with advancing age and presence of comorbid health conditions. Cancer patients are regarded as a highly vulnerable population to infection and development of more severe symptoms, which is possibly due to weakened immune systems, caused directly by tumor growth and indirectly by effects of anticancer treatment. However, there is scarce evidence about the impact of COVID-19 infection in patients with suspicion or a history of thyroid cancer. Indeed, early data from China suggest that COVID-19 does not worsen the outcome of cancer, but as the pandemic continues, with investigations and treatment delay, morbidity and mortality from thyroid cancer may increase (1, 6). In this review, we discuss some clinical challenges that clinicians could consider during this pandemic and we aim to update the clinical management guidance for patients with nodules and malignant neoplasms of the thyroid gland.

## How will COVID-19 impact patients with thyroid nodules and cancer?

Reports from China and the United States describing clinical, demographic characteristics and outcomes of patients with cancer and COVID-19 examined risk factors for mortality, documenting a higher rate of death due to COVID-19 and severe outcomes in patients with cancer. Despite thyroid cancer’s indolent nature, more recent studies have shown thyroid cancer as a frequent diagnosis among patients admitted to hospitals with COVID-19. However, these results are preliminary, and no causal association has yet been documented among thyroid cancer and COVID-19 infection. They are mainly attributed to the high prevalence of thyroid cancer and nodules (7, 8). To the best of our knowledge, no tumor-, patient- or treatment-specific factors conveying the increased vulnerability of infection have been identified yet. As a matter of fact, patients with thyroid cancer who have previously received disease-specific treatment such as surgery (with or without ^131^I administration), as well as the vast majority of those with suspicious thyroid nodule(s) (TNs) or neck lymphadenopathy, are not considered at higher risk of viral infection. Of course, there are some exceptions including transient reduction in myeloid and lymphoid cell lineages after ablative therapy which can last up to 1 year, as well as bone marrow suppression following prolonged high dose of radioiodine which has been described, and may increase the risk for COVID-19 severity and complications (9). On the contrary, patients who are receiving multikinase inhibitors, or chemotherapy, or patients who have previously received external beam radiotherapy to the neck are at increased risk of severe illness from coronavirus (10). Patients with stage IV disease with severe lung metastatic disease are also considered at higher risk for severe infection (https://www.btf-thyroid.org/thyroid-cancer-and-coronavirus, Accessed: June 4, 2021).

Viruses are thought to be the etiologic agents in approximately 20% of human cancers. Tumorgenesis in individuals with viral infection can take many years after infection, demonstrating that the involvement of viruses in cancer development is a long and complex process. Viruses associated with human cancers ensure their survival and proliferation through the activation of several cellular processes including inflammation, migration and invasion, resistance to apoptosis and growth suppressors. Oncogenesis and disease progression in thyroid gland have been as well associated with viral infections. Although preliminary and limited, recent data suggest a mechanism by which SARS-CoV-2 infection may contribute to oncogenesis. Policard* et al*. identified genes modulated by COVID-19 infection that are implicated in oncogenesis, including E2F transcription factors and RB1, laying the foundations for further research. Although recent data indicate that COVID-19 infection stimulates inflammatory–immune responses in thyroid gland, and extensive injury to the follicular epithelium and parafollicular cells has been described, there is no evidence yet associating SARS-CoV-2 with tumorigenesis in thyroid gland (11).

### A) How will COVID-19 impact the diagnosis of thyroid nodules?

Thyroid nodules are among the common diseases of the endocrine system, with 3–7% prevalence by palpation and 19–67% by high-resolution ultrasonography, showing annual increasing trends worldwide, while only 5–15% of TNs turn to be malignant (12, 13, 14). Therefore, differential diagnosis is critical for guiding clinical management. The clinical management of TNs needs to be multidisciplinary. Laboratory and radiologic evaluation play an integral part in the diagnostic algorithm, with ultrasonography (US) and fine-needle aspiration (FNA) biopsy playing a pivotal role in the diagnostic workup (15, 16, 17, 18). Other imaging modalities including CT, MRI, ^99m^Tc/^131^I/^123^I/^99m^Tc-SESTAMIBI scintigraphy and PET/CT are applied to a lesser extent especially during the pandemic (19, 20, 21). There is little evidence that early detection and treatment of DTC significantly alter disease outcomes, as the overall mortality rate for DTC has remained low at ~0.5%, despite a steady rise in its incidence, as neck imaging has become more widespread. Delays, postponements of key diagnostic procedures or alternative consultation must be performed after a careful balancing of the relative merits vs the potential risks of viral infection. Several authors have suggested and performed postponements of diagnostic fine needle aspiration biopsy (FNAB) and surgeries mainly during more restrictive quarantine phase.

The urgency of the FNA should be determined by the patient’s risk factors, the sonographic and structural risk characteristics of the nodule and the clinical judgment of the treating team. Timing of FNA may also be impacted by local public health directives on societal reopening. Moreover, it should be performed ‘safely’ without undue increase in risk of exposure to the patient, staff or the clinician to COVID-19 (https://www.thyroid.org/covid-19/clinical-committee-physician-guidance/, Accessed: June 23th, 2021). FNA of most asymptomatic TNs with no history of neck irradiation, with no familial history of thyroid cancer or multiple endocrine neoplasia syndromes, with favorable clinical and sonographic characteristics (using an acceptable scoring such as thyroid imaging reporting and data system (TIRADS)), without suspicion of medullary thyroid cancer (MTC) (e.g. with family history of MTC) or anaplastic thyroid cancer (ATC) and without compressive symptoms due to thyroidal masses is recommended to be postponed and rescheduled during restrictive quarantine phase (1, 22).

Taking into consideration the interaction between thyroid neoplasms and COVID-19 arising from the data that have been published so far, we recommend the following:

- Postponement and rescheduling or teleconsultation of scheduled outpatient clinical examination/imaging/functional test (US, ^99m^Tc/^123^I/^131^Iscintigraphy with or without % uptake) and FNA cytology for diagnostic or follow-up reasons for patients with suspicion or history of TNs or thyroid cancer, during the acute phase of the pandemic. Exceptions are:
  - patients with significant symptoms, indicating critical events (e.g. pressure to trachea and breathing difficulties) suggesting large goiter should undergo imaging for further assessment (always with precaution and risk/benefit assessment);
  - patients with history, clinical characteristics and laboratory examinations indicating aggressive thyroid disease, for example, anaplastic, medullary, metastatic or other diseases, for example lymphoma;
  - pediatric patients with non-incidental cervical findings;
  - high-risk patients (according to the American Thyroid Association (ATA) 2015 risk stratification system for recurrence in thyroid cancer patients (15)) and all patients with biochemical incomplete, structural incomplete or indeterminate response;
  - patients with local disease possibly infiltrating the trachea or the esophagus, or suspicious liver, brain or bone spread;
  - patients who have been fully vaccinated.
  All the aforementioned cases should be prioritized for clinical, laboratory and imaging evaluation during the pandemic, always with precautions and risk/benefit assessment. A preventing protocol with RT-PCR test 2–3 days before or same day rapid SARS-CoV-2 antigen detection test and questionnaire with suggestive COVID-19 symptoms and social isolation should be proceeded before any diagnostic or follow-up procedure.
- In periods during the pandemic that a decline in the number of newly diagnosed COVID-19 infections, ICU admissions and disease-related deaths is documented, all the clinical, laboratory and imaging work-ups for patients with TNs or neoplasms are recommended to be performed, using a preventing–screening protocol as previously mentioned.

### B) How will COVID-19 impact therapy for thyroid nodules and thyroid cancer?

Cancer patients are regarded as a highly vulnerable group in the current COVID-19 pandemic, showing deteriorating conditions, higher risk for ICU admission and poor outcomes from the infection. However, we need to acknowledge that malignant diseases are heterogenous and divided into different groups, especially according to cancer type, stage and clinical profile of each patient. Concerning thyroid cancer (TC) and COVID-19 outcomes and mortality, there are scarce published data. Among them, Sahin* et al.* showed that age, presence of diabetes mellitus, asthma/chronic obstructive pulmonary disease (COPD), heart failure, chronic kidney disease, prior coronary artery disease, renin–angiotensin–system blocker usage and low lymphocyte were positively associated with mortality in patients with TC and COVID-19 infection. Moreover, they showed that radioactive iodine (RAI) treatment and cumulative iodine activity did not negatively affect the severity and mortality of COVID-19 in this patient group.

The minority of TC patients with advanced or metastatic disease who are receiving multikinase inhibitors (such as lenvatinib or sorafenib), chemotherapy or external beam radiotherapy are at an increased risk of developing adverse events (with their prevalence ranging from 87 to 100%), resulting in a high probability of severe illness from SARS-CoV-2 (https://www.eurothyroid.com/news/covid-19-thyroid-diseases.html, Accessed: June 7, 2021). All the aforementioned cases that are at a higher risk of severe/critical infection with COVID-19, including patients with comorbidities, should be shielding and self-isolating. They should systematically follow general principles to help prevent the spread of airway and chest infections, including handwashing and respiratory hygiene and register for support when needed (https://www.thyroid.org/covid-19/statement-covid-19/, Accessed: June 15, 2021, https://www.gov.uk/government/publications/guidance-on-shielding-and-protecting-extremely-vulnerable-persons-from-covid-19/guidance-on-shielding-and-protecting-extremely-vulnerable-persons-from-covid-19/, Accessed: June 20, 2021). Moreover, acknowledging specific clinical characteristics of patients with malignant neoplasms as well as the risk for severe COVID-19 infection, the National Comprehensive Cancer Guidelines prioritize COVID-19 vaccinations for patients with cancer. For patients with solid tumor malignancies, the panel recommends vaccination, even if the patients are receiving cytotoxic chemotherapy, targeted therapy, checkpoint inhibitors and other immunotherapy or radiation (https://www.nccn.org/covid-19/, Accessed: June 29, 2021).

Taking into consideration the interaction between thyroid neoplasms and COVID-19 arising from the data that have been published so far, we recommend the following:

- During the acute phase of pandemic, postponement of all non-urgent surgery, even those for cytologically confirmed differentiated thyroid cancer. Exceptions are:
  - patients with large goiter causing regional critical for life events (e.g. pressure to trachea and breathing difficulties) or with rapidly growing TNs/ cancer;
  - patients with history, clinical characteristics and laboratory examinations indicating aggressive thyroid disease, for example, anaplastic, medullary, metastatic or other diseases, for example lymphoma;
  - pediatric patients with worrisome rate of progression (e.g. children of younger age or intermediate or high ATA pediatric risk for recurrence level) (23, 24);
  - patients who have been fully vaccinated.

All the aforementioned cases should be prioritized for (total) thyroidectomy and lymph node neck dissection during the pandemic, always with precautions and risk/benefit assessment. A COVID-19 prevention protocol should be followed during surgery (25, 26). Patients should also respect social distancing pre- and post-operatively, while avoiding visitors during hospitalization period is a sine qua non.

- In less acute pandemic period, all surgeries are recommended to be performed, using a strict preventing–screening protocol as previously mentioned since recent data have shown that head and neck surgery during the pandemic is safe even when prolonged and complex.
- Postponement of RAI (^131^I) therapy, either as remnant ablation or as adjuvant treatment (as defined in the Martinique principles (27)), especially for patients with comorbidities that are at higher risk for more severe COVID-19 infection (27). The need for repeated patient exposures to medical staff must be balanced against the perceived benefits of treatment. Moreover, the stage of the disease as well as the clinical profile of each patient should be evaluated, in order to avoid exceptional circumstances such as the need for hospital or ICU admission of a patient that has received a substantial dose of radioiodine. Exceptions are:
  - ^131^I therapy for which a patient has already begun pre-treatment such as administration of redifferentiating agents or thyroxine withdrawal.
  - Patients who have been fully vaccinated.
  All the aforementioned cases should be prioritized for radioiodine therapeutical administration during the pandemic, always with precautions and risk/benefit assessment. A preventing protocol should be proceeded before any RAI treatment procedure.

## How to manage acutely unwell patients without full nuclear medicine investigation and therapy?

Multidisciplinary approach is considered an indispensable condition for achieving safe and effective management of acutely unwell patients. Emergencies related to thyroid gland diseases are infrequently observed in clinical practice . TN/cancer-related emergencies are caused by overt dysfunction of the gland, which are so severe that require admission to ICU frequently. They include compressive symptoms caused by huge goiter and aggressive thyroid tumors, hypothyroid coma and thyrotoxic storm. MTC and ATC, as well as extensive metastatic disease of DTC, should also be considered as cases that require early diagnosis and prompt treatment, since they are characterized with unfavorable prognosis.

The diagnosis of goiter-related compressive phenomena is based on symptoms and signs complained by patients. Imaging examinations either confirm the clinical suspicion or show the surgeon goiter’s extension toward the neighboring structures, and particularly cleavage plans. The clinical picture is characterized by dyspnea with hypoxemia and respiratory acidosis, stridor, dysphonia, dysphagia and difficult or impossible extension/flexion of the neck. Rarely, superior vena cava syndrome may occur. Except for radioiodine or ^99m^Tc SPECT/CT scintigraphy or PET/CT, which only serve as adjunctive imaging methods in the diagnostic algorithm of goiter-related compressive phenomena, no other NM modalities could be of additional value in the diagnostic algorithm of such cases. Acknowledging the exceptional conditions under which a patient with severe compressive symptoms should be managed in the COVID-19 era, we recommend that SPECT and PET/CT should be substituted by other imaging modalities that do not include the administration of radiopharmaceuticals and are less time-consuming. Urgent surgery represents the gold standard treatment in patients with rapidly increasing neck masses associated with stridor with resultant morbidity. Patients requiring notwithstanding surgical intervention include those with evidence of aerodigestive tract compromise/invasion; recurrent laryngeal nerve palsy due to malignancy; locoregional metastasis; large, compressive tumors; clinical concern for example, rapid growth, poorly differentiated tumors; MTC or ATC. A preventing protocol should be proceeded with any surgery. However, in case of severe life-threatening symptoms, patients can be excluded from the preventing protocol.

## How should current patients with thyroid nodule/cancer be advised about risk? Who still needs to be seen and why? What/who can be converted safely to remote review?

Most TNs are benign, while malignant neoplasms are characterized by an indolent natural history. In this respect, most nuclear thyroidology (diagnostic and therapeutic) procedures are not urgent. There is little evidence that early detection and treatment of DTC significantly alter disease outcomes, which are not the case for ATC and MTC that need to be managed without delay. Therefore, for most cases, it is generally safe to delay diagnostic and therapeutic procedures including thyroidectomy and RAI treatment. However, it is vital for higher-risk cases to be early recognized and treated properly. Risk stratification as proposed by the latest ATA guidelines, TIRADS as well as dynamic clinical evaluation is a sine qua non, to ensure that patients with characteristics that imply an unfavorable prognosis, will be prioritized for diagnostic and therapeutic procedures during the pandemic. Very low, low and intermediate risk for recurrence patients can safely postpone diagnostic and therapeutic protocols, as described above. Personalized risk assessment should be performed to justify a delay in surgery even for high-risk patients with thyroid cancer. Except for local COVID-19 prevalence, tumor aggressiveness expressed as rapidly progressive disease, big invasive regional metastatic lesions, distant metastases or suspicion of tumor dedifferentiation should be taken into consideration. Priority for urgent intervention is placed on the need for control of high-volume neck disease and airway protection (7). A strict preventing protocol should be applied in any case to avoid or minimize the dispersion of SARS-COV2 virus.

After an individual risk–benefit analysis, diagnostic and therapeutic interventions may proceed for selective cases, despite the threat of COVID-19 infection (as stated in the previous section). All remaining patients can be safely reviewed remotely based on risk, in predefined time intervals, with an ‘open line’ for the patient to report abnormalities. Exceptions are fully vaccinated patients who can safely undergo diagnostic and therapeutic workup without delay. Proper remote consultation should be carefully planned since it is time-consuming. *A priori* patient notification is useful offering time for better preparation of possible questions to be referred. A convenient tool for institutions is to offer online-specific frequently asked questions on issues of possible patient information concerning, for example the risk of RAIT or surgery postponement. Numerous online information from different societies/groups specifically relating to COVID-19 and TNs and cancer are currently available, where patients could be referred to (1) (https://www.endocrinology.org/clinical-practice/covid-19-resources-for-managing-endocrine-conditions/, Accessed: June 26, 2021, https://thyroidpatients.ca/2020/03/29/questions-about-thyroid-and-covid-19-risk/, Accessed: June 25, 2021, https://thyroiduk.org/about-thyroid-uk/position-statement-covid-19/, Accessed: June 19, 2021, https://pro.aace.com/recent-news-and-updates/aace-position-statement-coronavirus-covid-19-and-people-thyroid-disease, Accessed: June 20, 2021, https://www.btf-thyroid.org/news/thyroid-disease-and-coronavirus-covid-19, Accessed: June 17, 2021, https://www.thyroid.org/covid-19/coronavirus-frequently-asked-questions/, Accessed: June 28, 2021).

## How should nuclear thyroidology services be remodeled in acute crisis?

The challenge not only for NM but also for all medical specialties during the pandemic is to ensure essential services are provided while keeping the patients and staff as safe as possible by reducing the potential exposure to COVID-19. Several reports, reviews, protocols and guidelines have been issued since the onset of the pandemic in the past year. Clinical practice has been altered, remodeled and adapted to the new circumstances in the COVID-19 era. The main adjustments that nuclear thyroidology has adopted are summarized into the following: general workplace adjustments including remote work for NM staff (at least for a group of them); postponing appointments for consultation, diagnostic and therapeutic purposes and rescheduling based on individualized risk stratification; telemedicine; preparation for possible issues on radiopharmaceuticals synthesis and delivery, preventing measures and protocols to minimize or avoid potential COVID-19 infection of patients and medical staff (28, 29, 30). The current COVID-19 pandemic has brought to light the need to apply focus on infection prevention in practice and update knowledge and procedures on such measures while ensuring both prognosis and quality of life for patients with TNs or cancer, always with a strong emphasis on radiation safety principles.

In addition to these suggestions, treating physicians should bear in mind the possibility that a treated patient could potentially be infected before, during or after hospitalization. Thus, the patient may require further specialized/acute care within or outside radioprotected areas (i.e. ICU) after discharge. Therefore, and to the extent possible, discharge rules should be applied more strictly and according to national rules (i.e. the discharge rules for the allowed residual radioactivity in each country should be strictly followed and, if meaningful, patients should be discharged with even lower levels), so that other healthcare practitioners and the general public remain protected in such a scenario, as already stated in our original paper.

### Disclaimer

Due to the emerging nature of the COVID-19 crisis, this document is not based on extensive systematic review or meta-analysis but on rapid expert consensus. The document should be considered as guidance only; it is not intended to determine an absolute standard of medical care. Healthcare staff needs to consider individual circumstances when devising the management plan for a specific patient.

## Declaration of interest

The authors declare that there is no conflict of interest that could be perceived as prejudicing the impartiality of this guidance.

## Funding

This work did not receive any specific grant from any funding agency in the public, commercial or not-for-profit sector.
